# Supplementary material for: Progress and gaps in childhood immunization among two-year-olds in Ghana (1993–2022): A trend and equity analysis
Source: PLoS One. 2026 Jun 5;21(6):e0349584. doi: 10.1371/journal.pone.0349584 (PMC13241012; doi:10.1371/journal.pone.0349584)
Supplement: S1 Table — (DOCX) [file pone.0349584.s001.docx]

# S1 Table. Administrative regions of Ghana and their geographical zones and characteristics.

| No. | Region | Geographical Zone | Key Characteristics |
| --- | --- | --- | --- |
| 1 | Greater Accra | Southern | The smallest but most urbanized region; includes the national capital (Accra) and highest population density. |
| 2 | Ashanti | Middle Belt | One of the most populous and economically vibrant regions; major trade and health hub (Kumasi). |
| 3 | Eastern | Southern | Mixed rural-urban region; important for agriculture and mining. |
| 4 | Central | Southern | Coastal region with fishing and tourism; moderate health service access. |
| 5 | Western | Southern | Coastal and resource-rich; known for oil and mining. |
| 6 | Western North | Southern | Recently created (2019); largely rural with farming communities. |
| 7 | Volta | Southeastern | Diverse ethnic composition; coastal and inland areas; moderate service access. |
| 8 | Oti | Northern part of Volta area | Carved from Volta Region (2019); mostly rural with limited healthcare access. |
| 9 | Bono | Middle Belt | Carved from Brong-Ahafo; semi-urbanized and agriculturally active. |
| 10 | Bono East | Middle Belt | Rural and agricultural; lower access to health facilities. |
| 11 | Ahafo | Middle Belt | Small, rural, with growing mining and agricultural activities. |
| 12 | Northern | Northern | Predominantly rural; high poverty levels; limited health infrastructure. |
| 13 | North East | Northern | Carved from Northern Region (2019); low population density and healthcare access. |
| 14 | Savannah | Northern | Also created in 2019; least urbanized and sparsely populated. |
| 15 | Upper East | Northern | One of the poorest regions; predominantly rural with limited health coverage. |
| 16 | Upper West | Northern | Least populated region; rural and characterized by low healthcare access. |
