# Supplementary material for: Progress and gaps in childhood immunization among two-year-olds in Ghana (1993–2022): A trend and equity analysis
Source: PLoS One. 2026 Jun 5;21(6):e0349584. doi: 10.1371/journal.pone.0349584 (PMC13241012; doi:10.1371/journal.pone.0349584)
Supplement: Attachment — Submitted filename: Response to Reviewers Comments_R1.odt [file pone.0349584.s003.odt]

RESPONSE TO REVIEWERS’ COMMENTS

2. Thank you for uploading your study's underlying data set. Unfortunately, the repository you have noted in your Data Availability statement does not qualify as an acceptable data repository according to PLOS's standards.
At this time, please upload the minimal data set necessary to replicate your study's findings to a stable, public repository (such as figshare or Dryad) and provide us with the relevant URLs, DOIs, or accession numbers that may be used to access these data. For a list of recommended repositories and additional information on PLOS standards for data deposition, please see https://journals.plos.org/plosone/s/recommended-repositories.
Response
We thank the reviewer for this important comment. We acknowledge that the previously cited WHO HEAT platform is not an acceptable data repository under PLOS ONE requirements. In response, we have now deposited the minimal analytical dataset required to replicate our findings in a public repository (Figshare), and a DOI has been provided in the revised manuscript. The Data Availability Statement has been updated accordingly.
3. Please ensure that you refer to Figure 1 in your text as, if accepted, production will need this reference to link the reader to the figure.
Response
We thank the reviewer for this helpful suggestion. We have ensured that Figure 1 is referenced in the main text to facilitate proper linking during production.

4.We note you have included a table to which you do not refer in the text of your manuscript. Please ensure that you refer to Table 2 and 3 in your text; if accepted, production will need this reference to link the reader to the Table.
Response:
We thank the reviewer for this helpful suggestion. We have carefully revised the manuscript to ensure that all tables, including Tables 2 and 3, are consistently cited in the main text to comply with journal production requirements.
5. Please include captions for your Supporting Information files at the end of your manuscript, and update any in-text citations to match accordingly. Please see our Supporting Information guidelines for more information: http://journals.plos.org/plosone/s/supporting-information.
Response:
We thank the reviewer for this helpful comment. We have revised the manuscript to include a properly formatted caption for Supporting Information (S1 Table) in accordance with PLOS ONE guidelines. We have also ensured that the Supporting Information file is correctly cited in the main text for clarity and consistency.

6. If the reviewer comments include a recommendation to cite specific previously published works, please review and evaluate these publications to determine whether they are relevant and should be cited. There is no requirement to cite these works unless the editor has indicated otherwise. 
Response: 
We acknowledge the editor’s guidance regarding citation of reviewer-suggested literature and have evaluated all suggested references for relevance. Only those deemed directly relevant to the study have been incorporated.

Reviewer #1: 
This is a nicely written article trying to identify some associations between low vaccination coverage and equity. The authors have used the HEAT database that is publicly available. However, they should consider the following points.
1. Although the authors have given the PAR and PAF in Table 3, they have given no description or interpretation of these two measures in either the Results or the Methods sections. They should explain to the reader the utility and practical applications of these measures in the context of Ghana.
 Response 
We thank the reviewer for this important comment. We have now clarified the description and interpretation of the Population Attributable Risk (PAR) and Population Attributable Fraction (PAF) in the manuscript. Specifically, we have ensured that their definition and interpretation are clearly stated in the Methods section, and we have added further explanation in the Discussion section to highlight their utility in quantifying the population-level impact of inequality and their relevance for informing equity-focused immunization strategies in Ghana. These revisions have been incorporated into the revised manuscript.

2. In Table 1 they should add what “mu” stands for. I have assumed it represents the weighted sample mean.
Response
We thank the reviewer for this helpful suggestion. We have now clarified the definition of “μ” in Table 1 by adding a footnote specifying that it represents the overall weighted mean immunization coverage in the study population. This clarification has been included in the revised manuscript
3. The equity analysis is based on grouped data and the putative determinants or explanatory variables of inequitable coverage (like socio-economic status, subnational area etc.) as well as the response variable are all group average values. A key limitation of such analyses is the group correlation fallacy or ecological fallacy in epidemiology, because the within group variation of explanatory variables are subsumed within the group average values. The authors should mention this in their discussion on limitations.
Response 
We thank the reviewer for this observation. We agree that, since our equity analysis is based on grouped data and group-level averages for both explanatory variables and outcomes, there is a potential risk of ecological (group correlation) fallacy, as within-group variability is not captured in the analysis. We have now acknowledged this as a limitation in the Discussion section, noting that interpretations are based on aggregate-level associations and may not fully reflect individual-level relationships. This clarification has been added to the limitation session of the manuscript. 

4. Discussion on some practical use of this research to the programme manager will enhance the usefulness of this manuscript. Authors can reflect the proportion of population affected by a given measure of inequity. For instance, of the two subnational areas with the lowest coverage in 2022 – Northern and Upper East – Northern has ~11% of the population whereas Upper East has only about 5% of the population. Discussing the equity analyses in this context will help the programme manager decide on prioritizing intervention to achieve the maximum impact.
Response
Thank you for this insightful suggestion. We have revised the Discussion to incorporate the practical implications of equity analyses for programme prioritization. Specifically, we now consider population distribution alongside inequality measures. Using data from the Ghana Demographic and Health Survey 2022, we highlight that regions differ in population size (e.g., Northern 7.6% vs Upper East 4.2%), which has implications for maximizing the impact of targeted interventions.

5. In the tables the decimal points for coverage estimates are cluttering up the display. Authors and copy editors may consider rounding off the decimal points.
Response
Thank you for this helpful suggestion. The coverage estimates in the tables are already presented to one decimal place to ensure a balance between readability and precision. We have retained this level of rounding, as the values are weighted estimates and further rounding may reduce accuracy. We hope this presentation is acceptable.

Reviewer #2: 
This important and interesting study, conducted under the auspices of various public organizations, including the WHO, assesses the effectiveness of vaccination coverage among children in their first years of life in Ghana. While it is interesting and logically written using appropriate statistical methods, it is essentially presented as a report on the implementation of expanded programs on immunization in a specific region and will be more useful in WHO bulletins and other documents for organizing vaccination programs and identifying gaps. However, it has weak scientific significance. The only significant point I found in the presented manuscript was the fact that, over a long period of observation in this specific region, a gap in support for the group of adolescents, that renew annually, persists. Possible ways to overcome this problem, including examples from other countries, are needed to present this article's scientific significance.
Response
Thank you for this important suggestion. We have revised the Discussion section to strengthen the scientific contribution of the manuscript by expanding its relevance beyond Ghana. Specifically, we now highlight that the observed inequalities are consistent with evidence from other low- and middle-income countries, with supporting examples from Kenya and Côte d’Ivoire. We also include a brief discussion of evidence-based strategies used in similar settings, such as prioritizing zero-dose children, strengthening outreach services, and integrating immunization with primary healthcare systems. In addition, we clarify the scientific value of the study by emphasizing the use of equity-focused measures (PAR and PAF), which provide a transferable framework for assessing and addressing immunization inequalities across comparable settings.
